# Long term treatment with ataluren—the Swedish experience

**DOI:** 10.1186/s12891-021-04700-z

**Published:** 2021-09-30

**Authors:** Eva Michael, Kalliopi Sofou, Lisa Wahlgren, Anna-Karin Kroksmark, Már Tulinius

**Affiliations:** 1 Department of Paediatrics, Region Västra Götaland, Sahlgrenska University Hospital, Gothenburg, Sweden; 2 Department of Paediatrics, Institute of Clinical Sciences, Sahlgrenska Academy, University of Gothenburg, Gothenburg, Sweden

**Keywords:** Duchenne muscular dystrophy, Ataluren treatment, Performance of upper limbs, Forced vital capacity, Long-term effect, Ambulatory vs non-ambulatory

## Abstract

**Introduction:**

Ataluren is a relatively new treatment for male patients with Duchenne muscular dystrophy (DMD) due to a premature stop codon. Long-term longitudinal data as well as efficacy data on non-ambulant patients are still lacking. Here we present the results from a long-term follow-up study of all DMD patients treated with ataluren and followed at the Queen Silvia Children’s Hospital in Gothenburg, Sweden, with focus on the evolution of patients’ upper motor and respiratory function over time.

**Methods:**

This is a retrospective longitudinal case-series study of all male DMD patients treated with ataluren and followed at the Queen Silvia Children’s Hospital in Gothenburg, Sweden, since 2008.

**Results:**

Our eleven patients had a median exposure to ataluren of 2312 days which is almost a fourfold higher than previous studies. Loss of ambulation occurred at a median age of 13.2 years. Patients who lost ambulation prior to 13.2 years of age had received ataluren for 5 years, whereas patients who continued to be ambulatory after 13.2 years of age had received ataluren for 6.5 years until loss of ambulation or last follow-up if still ambulatory. Four of six non ambulatory patients had Performance of the Upper Limb scores above the expected mean values over time. All but one patient maintained a pulmonary decline above the expected over time. All ambulatory patients increased in their predicted forced vital capacity (FVC) with 2.8 to 8.2% annually. Following loss of ambulation, 5 of 6 patients declined in predicted FVC (%), with annual rate of decline varying from 1.8 to 21.1%. The treatment was safe and well tolerated throughout the follow-up period.

**Conclusions:**

This is the first study to present long-term cumulative treatment outcomes over a median period of 6.3 years on ataluren treatment. Our results indicate a delay in loss of ambulation, as well as a slower decline in FVC and upper limb motor function even after loss of ambulation. We suggest that treatment with ataluren should be initiated as soon as the diagnosis is confirmed, closely monitored and, in case of sustainable benefit, continued even after loss of ambulation.

## Introduction

Duchenne muscular dystrophy (DMD) is an X-linked, progressive neuromuscular disorder mainly affecting males, with symptom onset around 2–3 years of age. It presents with proximal muscle weakness and pseudohypertrophy of the calf muscles, as well as very high levels of creatine kinase (CK) in the blood. Over the years there is progressive deterioration, with loss of ambulation between 7–13 years. The two main causes of death in early adulthood are cardiac and/or respiratory insufficiency [1, 2]. Currently, there is no cure for this disorder. Oral glucocorticoids have been shown to slow the disease progression but are not curative [3]. Along with early rehabilitation, they are the treatment cornerstones for this disorder. The rehabilitation includes physiotherapy, occupational therapy and psychosocial intervention. These are initiated as soon as the diagnosis is established and reevaluated 1–2 times per year, in order to help patients develop and maintain motor functions but also prevent development of contractures, scoliosis and other irreversible symptoms due to the disease progression [4].

In 10–15% of patients with DMD the cause is nonsense mutations leading to premature stop codons in the reading frame of the dystrophin gene, resulting in truncated dystrophin protein [5, 6]. In recent years a small non-aminoglycoside molecule, known as ataluren, has been developed which promotes readthrough of the premature stop codon in the mRNA and thus dystrophin expression [7, 8]. Studies have shown this to be a safe and effective treatment, that seems to slow down the disease progression if given early in the disease course [5, 8, 9]. Ataluren is now approved by the European Medicine Agency (EMA) for the treatment of male patients older than 2 years of age with DMD caused by a premature stop codon.

Ataluren is a rather new treatment and long-term longitudinal data are still lacking. In 2020, Mercuri et al. published data comparing ataluren treatment from the Strategic Targeting of Registries and International Database of Excellence (STRIDE) registry to standard-of-care glucocorticoid treatment alone, from the Cooperative International Neuromuscular Research Group (CINRG) Duchenne Natural History Study (DNHS) [5]. Their results showed a delay in symptom progression of DMD patients on ataluren compared to those on corticosteroids. As the STRIDE registry was initiated in March 2015, there is limited data on the long-term effects of ataluren on disease progression and more specifically on upper limb motor function as well as respiratory and cardiac function. The goal of this article is to present results from a long-term follow-up of our patients with DMD treated with ataluren, focusing mainly on how their upper motor as well as respiratory function has evolved over time.

## Methods

This is a retrospective longitudinal case-series study of all male DMD patients who have been treated with ataluren and followed at the Queen Silvia Children’s Hospital in Gothenburg, Sweden, since 2008. All these patients had a genetically verified nonsense mutation leading to a premature stop codon.

### Study population

**Fig. 1:**
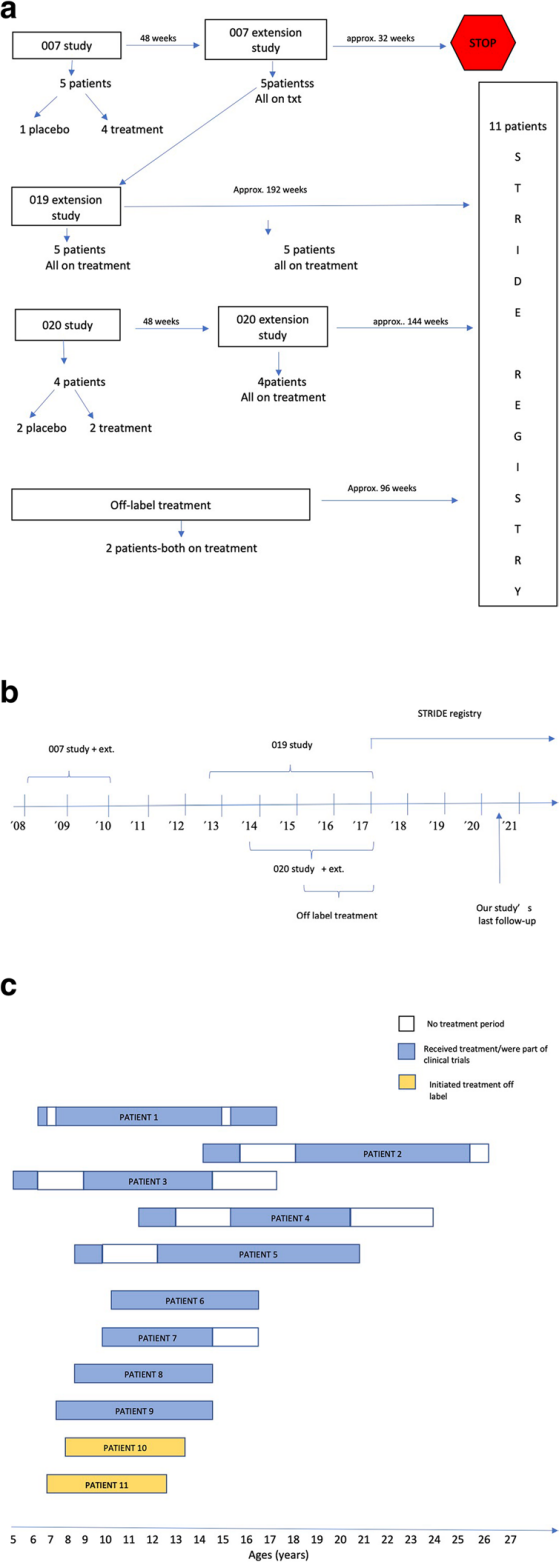
**a** Patient inclusion flowchart, showing how the number of patients in each clinical trial, how many were on placebo vs active treatment and the duration of each clinical trial until all subjects were included in the STRIDE registry. **b** timeline starting from 2008 when the first clinical trial with ataluren started until the current time period. **c** ataluren treatment in each patient, showing age at start of ataluren, duration of treatment (shown in blue or yellow colour) as well as periods without treatment (shown in white colour)

Most patients initially received ataluren as part of their enrolment in prospective, controlled clinical trials; the PTC 007 study, its extension study PTC 019 [9] and the PTC 020 study [10], as detailed in Fig. 1a and b. As part of these trials, patients’ treatment periods with ataluren varied, as shown in Fig. 1c. Five patients had ‘off-treatment’ periods between trials (Fig. 1c). Two patients did not initially receive ataluren as part of a clinical trial, but as off-label (Fig. 1a and c). Since 2017, all patients have been enrolled in the STRIDE registry. After treatment with ataluren was granted approval by the EMA in 2017, all patients continued to receive their treatment through their county hospital, having regular follow-ups at our clinic with the same array of tests, as part of the STRIDE registry follow-up protocol.

### Data collection

Upon initiation of ataluren treatment, all patients were followed in a prospective, systematic manner, either as part of their enrolment in a clinical trial (Fig. 1) [8–10] or as part of regular follow-ups to assure continued medical surveillance and disease monitoring. The follow-ups included physical examination and physiotherapeutic evaluation every 24 weeks, as described below. Since 2013, lung function tests were added to the follow-ups, twice per year. For the purposes of this study, data were retrospectively collected from the patients’ medical records using a case report form (CRF) which is available upon request. Data included age at symptom onset, age of corticosteroid start and dosage, muscle biopsy and genetic results, co-morbidities, other medications, hospitalizations and number of infections per year.

For the pulmonary and motor function tests, the time period 1^st^ January 2013 to 1^st^ November 2020 was used, to have a more homogenous set of data. Since 2017 all collected data are also part of the STRIDE registry database.

### Parameters performed and presented in our results

#### Physical examination

Physical examination including complete neurological examination and history taking since past visit were performed every 24 weeks by a pediatric neurologist [8–10].

#### Performance of the upper limb module

Assessment of upper limb function was performed with the Performance of the upper limb module (PUL 1.2) developed by Mayhew et al. [11]. The test was designed for ambulant and non-ambulant patients with DMD. The test comprises modules for shoulder, elbow and wrist and hand dimensions with a total score of 74 points. This function test was first introduced in 2013 for patients who have lost ambulation.

#### Lung function tests

Forced vital capacity (FVC) was measured with a spirometer (Vitalograph, Alpha, Model 6000, Ennis, Ireland and MasterScreen™ PFT System, Vyaire Medical inc, former Jaeger) in a sitting position. The best out of three trials was used for further analysis. The value, FVC%, was given as percentage of reference value correlated to sex, age and height [12].

### Statistical analysis

The statistical evaluations performed were mainly descriptive. Due to the small number of patients, frequencies were presented as median and minimum/maximum scores. Histograms and scatter plots were used to provide a visual of data distribution and illustrate differences among patients.

## Results

### Demographics

A total of 11 male DMD patients with a verified nonsense mutation in the dystrophin gene were identified. All were on daily corticosteroid treatment with a stable, weight appropriate dosage, starting at a median age of 4 years (3y-5y, Table 1). All patients had an individualized, daily physiotherapy program, with stretching exercises for both upper and lower limbs and usage of orthoses when needed, that were continued even during the clinical trials and the follow-up period. Patients’ genotype, disease-specific characteristics and treatment overview are summarized in Tables 1 and 2.

**Table 1:** Disease-specific characteristics and treatment overview for all our patients (*n* = 11)

**Demographics**	
**Characteristics**	
**Gender, n (%)**	
Male	11 (100.0)
**Age at symptom onset, years (*n* = 8; 3:not available)**	
Median	1.75
Min, Max	1.5, 4.0
**Age at diagnosis, years (*n* = 10; 1:not available)**	
Median	3.75
Min, Max	3.0, 5.0
**Age at last follow-up, years (*n* = 11)**	
Median	16.2
Min, Max	12.2, 26.45
**Ambulation status at last follow-up, n (%)**	
Ambulatory	4 (36.4)
Non-ambulatory	7 (63.6)
**Age at loss of ambulation, years (***n*** = 7; 4:Not Applicable)**	
Median	13.2
Min, Max	8.5, 18.1
**Age at start of ataluren treatment, years (*n* = 11)**	
Median	8.4
Min, Max	5.2, 14.4
**Cumulative use of ataluren, years (*n* = 11)**	
Median	6.3
Min, Max	4.0, 9.35
**Age at start of corticosteroid treatment, years (*n* = 11)**	
Median	4.0
Min, Max	3.0, 5.0
**On-going ataluren treatment at last follow-up, n (%)**	7 (63.6)
**On-going corticosteroid treatment at last follow-up, n (%)**	11 (100.0)
**Age at discontinuation of ataluren treatment, years (*n* = 4)**	
Median	17.6
Min, Max	14.2, 25.5

**Table 2:** Overview of patients main clinical and genetic findings as well as treatment used (*n* = 11)

**Patient ID**	**Age at onset**	**Corticosteroid treatment (Yes/No)/Type**	**Age at start of corticosteroid treatment (years)**	**Age at ataluren start (years)**	**Age at last follow-up (years)**	**Ambulation status at last follow-up**	**Age at loss of ambulation (years)**	**Cumulative use of ataluren (years)**^a^	**Age at discontinuation of ataluren (years)**	**Genotype**
p1	4	Y/P	4	6.2	17.4	Non-ambulatory	12.9	8.3	NA	exon 19, p.Lys773*
p2	n.a	Y/P	4	14.4	26.5	Non-ambulatory	17.1	8.6	25.5	Exon 30, p.Gln1370*
p3	1.5	Y/P	4	5.2	17.3	Non-ambulatory	12.4	6.9	14.7	exon 52, p.Arg2553*
p4	1.5	Y/D	4	11.5	23.3	Non-ambulatory	13.5	6.3	20.5	Exon 39, p.L1834X
p5	1.5	Y/P	4.5	8.4	20.3	Non-ambulatory	18.1	9.4	NA	exon 35, p.R1666*
p6	n.a	Y/P	Preschool^b^	10.3	16.2	Ambulatory	NA	5.9	NA	exon 20, p.Ser861*
p7	4	Y/D	5	10.2	16.0	Non-ambulatory	12.9	4.0	14.2	exon 58, p.Glu2886*
p8	2	Y/P	4.5	7.6	14.3	Ambulatory	NA	6.8	NA	exon 70, p.Arg3381*
p9	2	Y/P	3	8.8	14.0	Ambulatory	NA	5.3	NA	exon 33, p.Glu1540*
p10	1.5	Y/P	3	8.0	13.0	Non-ambulatory	8.5	5.0	NA	Exon 3, p.Leu1675X
p11	n.a	Y/P	3.5	6.9	12.2	Ambulatory	NA	5.3	NA	exon 70, p.Arg3381*

*Y* Yes, *P* Prednisolone, *D* deflazacort, *n.a.* not available, *NA* Not Applicable

^a^ Cumulative use of ataluren before loss of ambulation or last follow-up if ambulatory (years)

^b^ At preschool age, exact date unknown

*denotes the position of the premature stop codon

All patients had different age and disease duration when initiated on ataluren. They were clinically followed to a median age of 16.2 years (12.2y-26.45y). The patients started ataluren treatment at a median age of 8.4 years (5.2y-14.4y) and the median exposure to ataluren was 2312 days (1472d-3413d). Treatment with ataluren was discontinued in four patients, at a median age of 17.6 years (14.2y-25.5y), all were non ambulant at the time of termination.

One patient (p3) could not perform reliably on the pulmonary and motor function tests throughout the study due to neuropsychiatric disorder and was thus excluded from the analyses below. The results presented below refer to the remaining 10 patients.

### Motor function outcomes

Four of 10 patients were ambulatory at last follow-up. Loss of ambulation occurred at a median age of 13.2 years (8.5y-18.1y). Three patients who lost ambulation prior to 13.2 years of age had received ataluren for a median period of 5 years (4y-8.25y). Six patients who continued to be ambulatory after 13.2 years of age had received ataluren for a median period of 6.5 years (5.25y-9.35y) until loss of ambulation or last follow-up if ambulatory. One ambulatory patient was 12.2 years old at last follow-up (p11) and was therefore not included in the estimations above. Two patients (p2 and p4) lost ambulation while they were off-treatment between trials for 2.5 years. One patient (p10) started on ataluren off-label at 8 years of age and lost ambulation only after 6 months. This was the most severely affected patient of our cohort as shown in all motor and respiratory measurements below.

**Fig. 2:**
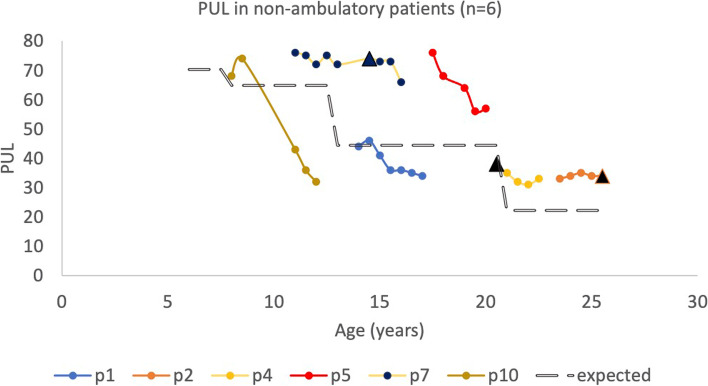
Change in PUL scores over time in non-ambulatory patients (*n* = 6). For patients 5,7 and 8, time at loss of ambulation is indicated with black triangle. In patients 1, 2 and 4 occurred previous to PUL examinations, at 12.9, 17.1 and 13.5 years old, respectively. The rate of expected decline in PUL shown with dot-line is adjusted by Pane et al. Neuromuscular disorders, 2014 [9]

PUL assessments were mainly performed in the non-ambulatory patients (*n* = 6). PUL change over time is summarized in Fig. 2. Only 3 of 6 patients had performed PUL assessment prior to loss of ambulation, as indicated in Fig. 2. Following loss of ambulation, 4 of 6 patients had PUL scores above the expected mean values over time (Fig. 2, [13]).

### Pulmonary function outcomes

**Fig. 3:**
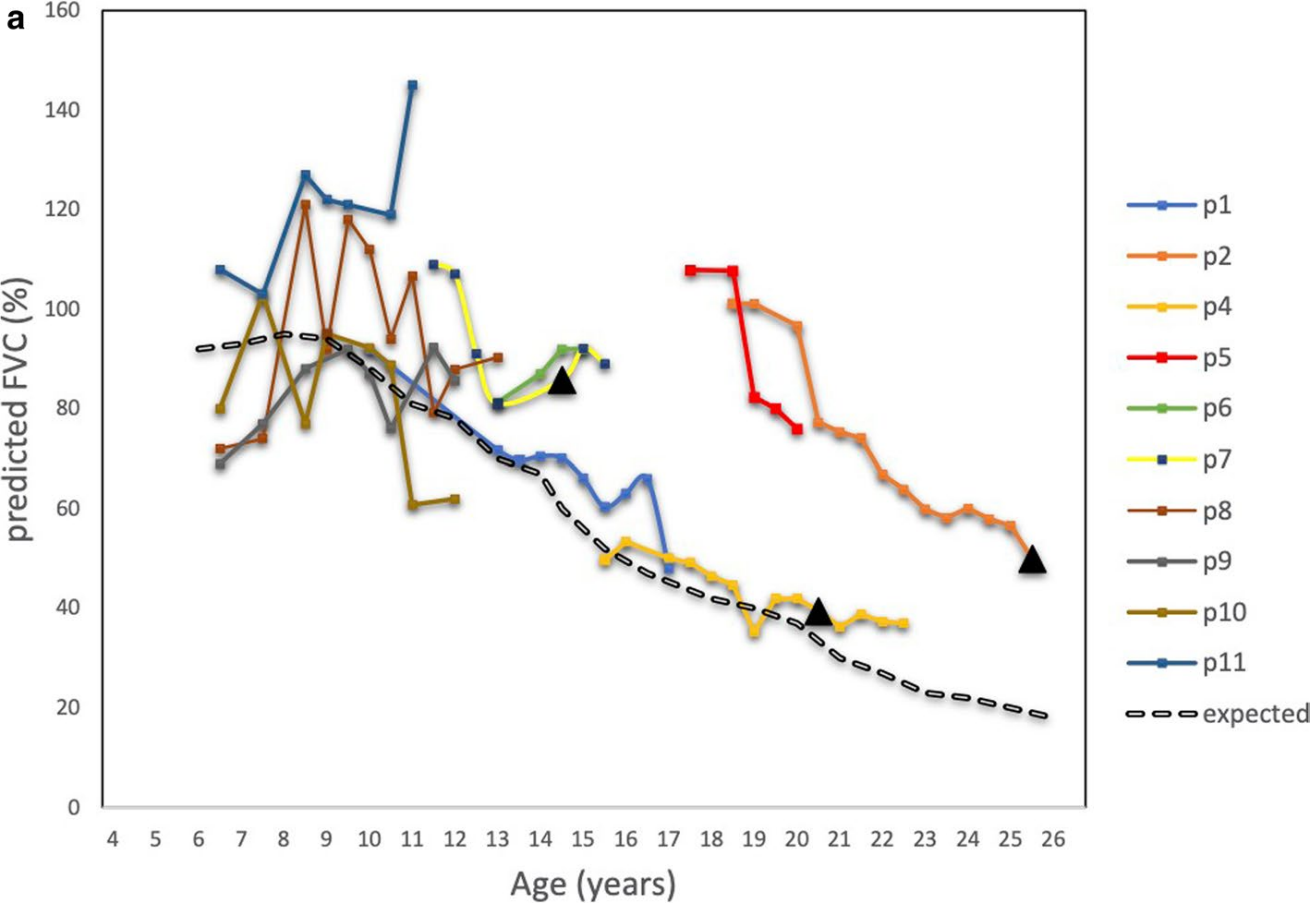
**3a** Change in predicted FVC (%) per patient over time (*n* = 10). The rate of expected change in predicted FVC (%) is adjusted by Mayer et al. 2015: Advances in pulmonary care in Duchenne Muscular Dystrophy [10]. **3b** Length of time periods on ataluren treatment per patient (*n* = 10). Age at discontinuation of treatment with ataluren is indicated with a black triangle for patients 2, 4 and 7

All patients except one (p10) maintained a pulmonary decline above the expected over time, as shown in Fig. 3a. Two of 10 patients declined in predicted FVC % lower than 50% (p1 and p4) at the age of 17 and 17.5 years respectively.

Two patients had nocturnal non-invasive ventilation at last follow-up and one patient had it at daytime for 30 min as part of his mucus mobilization regime. None of our patients had continuous ventilatory support. The rate of pulmonary decline as measured by predicted FVC% per patient over time, is shown in Fig. 3a (*n* = 10). The mean rate of expected pulmonary decline in FVC % is also depicted (Fig. 3a, [14]). The length of time periods on ataluren treatment per patient is shown in Fig. 3b.

FVC lower than 1 L was seen in one of 10 patients (p4), at the age of 19 years. This patient first received ataluren at the age of 11.5 years for a period of 1.5 years. The treatment was resumed at the age of 15.4 years and at that time the patient had predicted FVC 50%. Since then, the patient showed a pulmonary decline as per the expected decline (Fig. 3a) and the treatment was discontinued at the age of 20.5 years. During this 5-year period on treatment the FVC declined 0.19 L (from 1.1 to 0.9 L).

Following treatment discontinuation, no substantial changes in pulmonary function were seen (p4 and p7), within a follow-up period of 2 years and 1 year respectively (Fig. 3a and 3b).

### Pulmonary function outcomes: non-ambulatory vs ambulatory patients

**Fig. 4:**
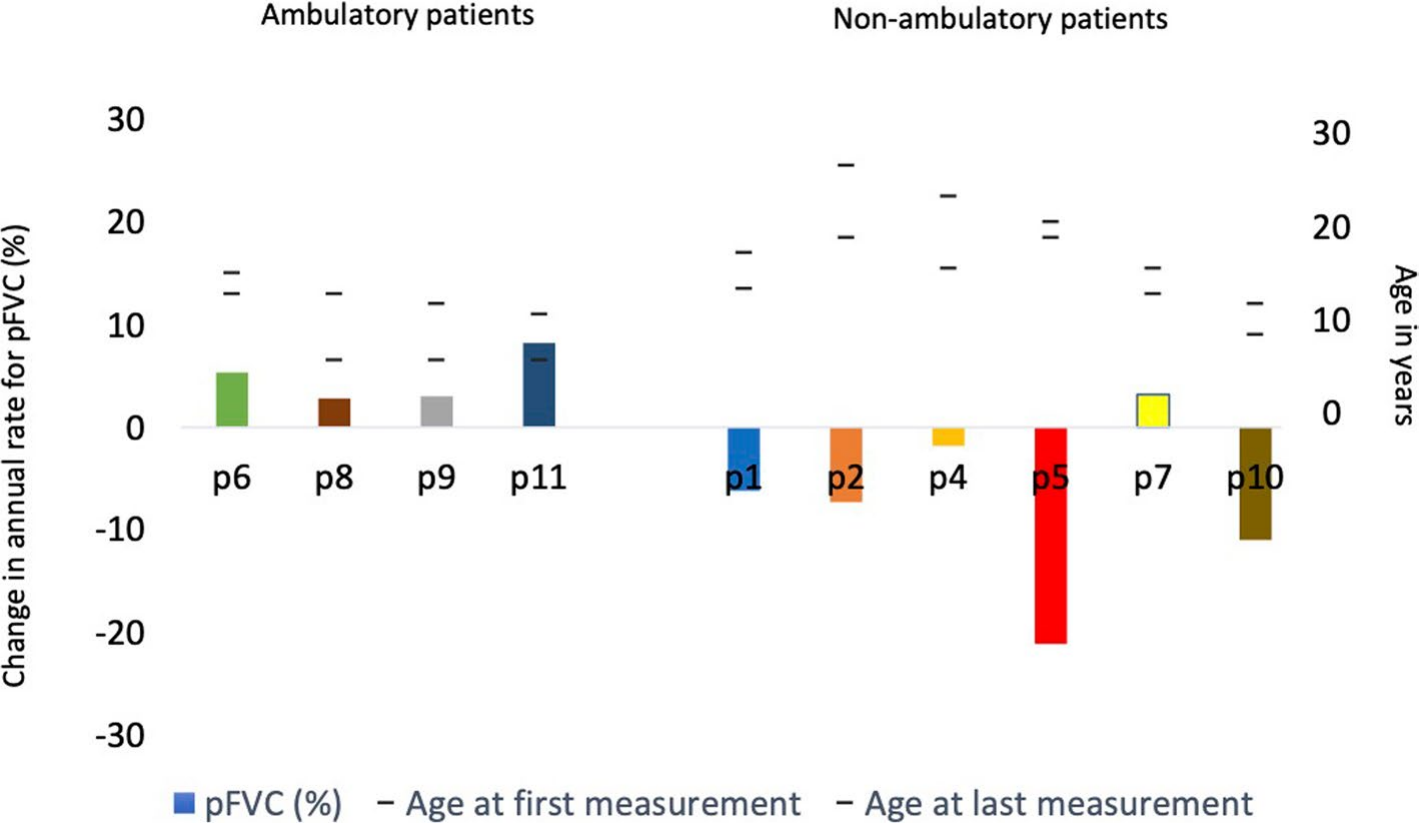
Annual rate of change in predicted FVC (%) for the non-ambulatory versus the ambulatory patients, as well as the respective age span (*n* = 10)

For the ambulatory patients, an increase in the predicted FVC % was seen with annual rates ranging from 2.8 to 8.2%. Following loss of ambulation 5 out of 6 patients declined in predicted FVC %, with annual rate of decline varying from 1.8% to 21.1% (Fig. 4). One patient had an increase in predicted FVC by 3.2%. The annual rate of change in predicted FVC % for the non-ambulatory versus the ambulatory patients, as well as the respective age span, are summarized in Fig. 4. Three patients (p2, p4, p7) had stopped treatment with ataluren by the last follow-up.

### Cardiac outcomes

None of the patients had developed acute or chronic heart failure by the last follow-up. Over the years all patients had regular cardiological evaluations and were started on cardioprotective treatment according to standard of care protocol for DMD, mainly angiotensin-converting enzyme inhibitor (ACEI) treatment [15].

Patient 4 showed decreased muscle contractility as well as a right bundle brunch block with tachycardia. He has therefore also been treated with metoprolol, spironolactone and digoxin. Follow-up with magnetic resonance imaging of the heart at 19 years of age showed good contractility and normal ejection fraction. Patient 5 developed hypertension and has been treated with both an ACEI and a calcium channel blocker.

### Safety

Ataluren was well tolerated with minor side effects. One patient had a low lymphocyte count and after discontinuation of ataluren treatment the values normalized again. He was reinitiated after 2 months at the same dosage without any recurrence. Another patient had microscopic blood in urine without signs of urinary tract infection. He stopped the medication and after normalization of the urine sample he was reinitiated without any recurrence. When asked during their regular follow-ups, all patients reported that, when ‘on-treatment’, they either felt generally better or didn’t experience any worsening of their motor function or other symptoms of disease progression over time.

Compliance to treatment was very high. Only one patient (p2), who was over 18 years old at the time, showed poor compliance due to lack of motivation over a 3-month period. This was discovered by our team and his family supported and motivated him to stay on treatment and receive ataluren as prescribed.

### Treatment termination

At last follow-up, three patients (p2, p4, p7) had terminated ataluren treatment. Once ataluren was approved by FDA and EMA, ataluren treatment was provided by each patient’s regional hospital and they continued with regular follow-up at our site. Since ataluren was recommended only for ambulatory patients, a discussion ensued, and these three non-ambulatory patients terminated ataluren treatment. Patients 2 and 4 were 25,5 and 20,5 years respectively when they terminated their treatment. Patient 7 was 14,5 years old when he stopped ataluren treatment because of continuing decline in upper motor function despite treatment.

## Discussion

Ataluren is a relatively new, promising treatment for DMD caused by premature stop codon mutations. We hereby present the Swedish long-term experience with ataluren. To our knowledge, this is the first study that presents long-term cumulative treatment outcomes over a median period of 6.3 years on ataluren treatment. Our results indicate a delay in loss of ambulation similar to the STRIDE registry study [5], as well as slower decline in FVC and in upper limb motor function. The treatment was considered safe and well-tolerated, while there were no treatment-related issues of non-compliance.

In our cohort ataluren was well tolerated with minor side effects, an observation consistent with previous descriptions for several other studies [5, 8–10, 16]. Our patients’ general impression of the treatment was positive and they stated that they felt both better and that their motor function was either unchanged or had improved. For them it was as important not to progress further as it was to improve in motor function.

Our cohort consisted of 11 boys of different ages and disease duration upon start on ataluren. Since 9 of 11 patients were part of randomized placebo-controlled clinical trials, some of them initially received placebo for 48 weeks before starting on active treatment. Our patients were initiated on treatment with ataluren at a younger age compared to those reported in the STRIDE registry (mean of 8.6y vs 9.8y respectively) and had an almost fourfold higher time of exposure to ataluren compared to the STRIDE registry [5, 17].

The median age at loss of ambulation of our patients was 13.2 years, which was later than in the CINRG DNHS study (mean 11 years) [5]. Since all of our patients were on a stable, weight-appropriate dosage of corticosteroids both at start and for the entire duration of ataluren treatment, we speculate that this delay could be due to ataluren; still, our cohort is little and our patients were of different ages and disease progression to be able to draw definitive conclusions. Nevertheless, this statement seems to be supported even by the STRIDE registry, where mean age at loss of ambulation was 14.5 years for those on ataluren treatment [5].

PUL was performed after loss of ambulation, so PUL results refer to a shorter time span compared to our other results. Four out of 6 patients (66.7%) had a change in PUL score over time that was superior to the expected decline suggested by Pane et al. [13]. Only 2 patients (p1 and p10) had values below the expected decline over time. One of these patients (p10) had the most severe disease in our cohort and progressed quickly over time. This is also observed by the steep decrease of his PUL scores compared to the other patients. The other patient (p1) was one of the patients who lost ambulation before initiation of PUL testing, at the age of 12.9 years. His PUL scores remained unchanged after 15 years of age until last follow-up at 17.4 years of age.

In 2015, Pane et al. presented even results of the 12-month change of the PUL score in DMD patients on corticosteroids, stratified to below or above 18 years of age [18]. In our 3 non-ambulatory patients over 18 years of age, we observed a 12-month change in their PUL score between -4 to + 2 points (+ 2, -4, -4 points), compared to a mean decrease of 3.13 points in the Pane et al. cohort. Our 3 non-ambulatory patients below 18 years of age had a 12-month decline between -1 to -11 points ( -1, -5, -11 points), compared to a mean decrease of 4.09 points in the Pane et al. cohort [18]. Patient 10, who was the weakest in our cohort, had a decline of 11 points. We believe our results to be in accordance to the result by Pane et al. despite our small cohort.

We propose that early initiation of Ataluren can be beneficial as it seems to lead to better ambulation outcomes. Our patients that started treatment at a young age (p6, p8, p9, p11) are still ambulatory at a median of 14.4 years of age. Patient 1 was 2.5 years off-treatment between trials and this may have contributed to a loss of ambulation at age 12.9 years. Patient 5 was also off-treatment between trials for 2.5 years, but he was 8.4 years old at treatment start. This patient has the highest cumulative treatment period in our cohort and this could have contributed to him being ambulatory until 18 years of age.

In 2015 Mayer et al. presented the predicted change in FVC over time for boys with DMD and calculated an annual decline of 5% points per year for ages 5 to 24 years [14]. In Fig. 3a we see the changes of FVC over time for our patients as compared to the predicted annual decline of FVC. All but one had a decline above that predicted by Mayer et al. Only two patients had an FVC below 50%, they were 17 and 17,5 years old respectively at that time which is higher than the median age of 16.9 years seen in CINRG DNHS study [5]. At the last follow-up only one of them had an actual FVC value below 1 L (pt4). This patient started ataluren treatment at an older age, had to discontinue treatment and was reinitiated at 15.4 years of age, by which time his FVC was already 50%. After reinitiating ataluren treatment and until his last follow-up his FVC has declined slowly (Fig. 3a).

When we compared ambulatory versus non ambulatory, the ambulatory patients had an annual increase in their predicted FVC % with a median of 4.19 per year (2.8–8.2%). At last follow-up these patients have had a median cumulative treatment period of 6.3 years.

In our non-ambulatory group, most patients declined over time but were still above the predicted decline according to Mayer et al. Only 1 patient (p10) showed values below the predicted decline and as mention he has been progressing fast despite treatment.

Three patients in our cohort had terminated ataluren treatment at last follow-up but the post-treatment period was short to draw any conclusions regarding the FVC changes post treatment. We speculate that the cumulative effect of ataluren could lead to higher FVC values before lung function begins to deteriorate and thus it will take longer to reach below 1L, delaying the age at which continuous ventilatory support is needed. This statement is strengthened by the fact that the majority of patients (9 of 10 pts) maintained a pulmonary decline above the expected over time.

Glucocorticosteroids (GCs) have been used since the late 80 s as part of the DMD treatment and it’s been established via clinical trials and the CINRG-DNHS study that early initiation of treatment leads to later loss of ambulation, later loss of upper limb milestones as well as better preserved FVC over the years, as compared to steroid-naïve patients [5, 19–21]. It is now recommended that DMD patients start GCs treatment early in the disease development, with most starting around 4—5 years of age, continuing even after the loss of ambulation and despite advanced disease progression. Termination of GCs usually occurs if the patient has very limited motor function left and has undesirable side effects of GCs [4].

Ataluren is a new medication and no consensus is established whether it should be continued once the patient loses ambulation. In our cohort, even after loss of ambulation, PUL values continued to be above the expected over time in the majority of patients (4 of 6 pts). As with FVC it could be speculated that deterioration of the upper limbs is delayed meaning that patients can maintain hand function over a longer period of time. It would be very interesting to investigate how the disease progression is affected if treatment with ataluren is initiated as soon as diagnosis is established but even continued after loss of ambulation and despite decline of upper motor function and respiratory function.

A limitation of our study is that we have a little cohort with a wide age span and the patients were in different disease stages both at the start and duration of the study. Most of our patients were part of different clinical trials and followed different study protocols making it difficult to have a homogeneous set of outcomes over a fixed time period for all the patients.

Another limitation is that there is no control group for our little cohort but we have instead used the CINRG-DNHS study and the STRIDE registry in order to compare our results.

Concluding, our 12-year experience of treating DMD patients with ataluren was positive. Treatment with ataluren seems to delay deterioration of clinically meaningful functional milestones, such as motor and pulmonary functions, while demonstrating a favorable safety and tolerability profile. Our results indicate that patients with DMD due to premature stop codon mutations may benefit of this treatment. They should be considered for ataluren treatment as soon as the diagnosis is established and continue treatment with close monitoring even after they lose ambulation as they may benefit in both pulmonary and upper limb motor function.

## Data Availability

All data generated or analyzed during this study are included in this published article.

## Abbreviations

DMD: Duchenne muscular dystrophy
CK: Creatinine kinase
EMA: European medicine agency
STRIDE registry: Stragetic Targeting of Registries and Databases of Excellence
CINRG- DNHS: Cooperative international neuromuscular research group- Duchenne natural history study
PUL: Performance of upper limb
FVC: Forced vital capacity
ACEI: Angiotensin converting enzyme inhibitor
CRF: Case report form
